# A heterogeneous graph attention network based IPv6 target generation algorithm for nonseed prefixes

**DOI:** 10.1038/s41598-026-52957-y

**Published:** 2026-05-22

**Authors:** Yangxiang Zhou, Liancheng Zhang, Haojie Zhu, Yakai Fang, Shunlong Hao, Jichang Wang, Wenhao Xia

**Affiliations:** 1 Information Engineering University, Zhengzhou, 450001 China; 2 Key Laboratory of Cyberspace Security, Ministry of Education, Zhengzhou, 450001 China; 3 School of Cyber Science and Engineering, Zhengzhou University, Zhengzhou, 450001 China

**Keywords:** IPv6 network, IPv6 address scanning, Pattern migration, Heterogeneous graph attention network, Nonseed prefixes, Computational biology and bioinformatics, Engineering, Mathematics and computing

## Abstract

The vast scale and sparse distribution of the IPv6 address space pose considerable challenges to efficient scanning. Current IPv6 target generation algorithms primarily rely on pattern mining of seed addresses to generate target addresses, which risks failing when encountering nonseed prefixes. To address this, researchers have proposed IPv6 target generation algorithms for nonseed prefixes. However, existing approaches suffer from limitations such as single-feature exploitation, inadequate utilization of Whois auxiliary information, and inability to handle missing Whois information, resulting in low target address hit rates and prefix coverage. Therefore, we propose 6HAN, which pioneers the use of a heterogeneous graph attention network to model multi-modal correlations. 6HAN first constructs a heterogeneous graph leveraging IPv6 prefixes and their Whois metadata. Subsequently, 6HAN integrates Whois auxiliary attributes with prefix structural features to generate prefix embeddings and designs a dual-task joint self-supervised framework for model training. Finally, 6HAN completes missing Whois fields through graph neural network-based correlation prediction, retrieves similar seed prefixes for pattern migration to form nonseed prefix address patterns, and generates high-quality IPv6 target addresses. Experimental results demonstrate that when sending 50 million probe packets, 6HAN improves the hit rates by 13.81%-201.05% and prefix coverage by 17.41%-233.58% compared with HMap6 and AddrMiner-N.

## Introduction

With the exponential growth in the number of global Internet users and devices, the depletion of IPv4 addresses has become increasingly critical^1^. As the core protocol of the Next-Generation Internet, IPv6 deployment and adoption have become an inevitable trend^2^. The vast IPv6 address space (extending $2^{128}$) presents unprecedented challenges for network mapping, security monitoring, resource management, and other fields. The core objective of IPv6 network space mapping is to detect active addresses efficiently. However, existing IPv6 target generation algorithms^3–7^ primarily rely on pattern mining from seed addresses. When confronted with nonseed prefixes (i.e. prefixes lacking known IPv6 seed addresses), these algorithms face the dilemma of having no seed address patterns to follow, severely limiting comprehensive understanding and security protection of IPv6 networks.

To address this, researchers have proposed a series of IPv6 target generation algorithms tailored for nonseed prefixes. Among these, HMap6^8^ employs a 4-bit granularity to traverse the IPv6 prefix space hierarchically. It selects low-byte or random addresses for preliminary detection within each subnet prefix, then uses clustering algorithms to mine active address patterns and generate target addresses. AddrMiner-N^9,10^ leverages Whois information similarity to associate nonseed and seed prefixes. It generates IPv6 target addresses under nonseed prefixes by migrating address patterns across prefixes.

The aforementioned algorithms effectively enhance the detection efficiency of active IPv6 addresses under nonseed prefixes, but they still exhibit the following limitations.

First, the feature utilization remains overly simplistic. For example, HMap6^8^ relies on low-entropy fixed bits or purely random generation strategies, focusing solely on the structural characteristics of IPv6 prefixes while neglecting semantic Whois attributes such as maintainer, network name, and service keywords.

Second, insufficient utilization of Whois auxiliary information. Algorithms such as AddrMiner-N^9,10^ involve keyword-matching-based pattern migration, but they only treat organization and country as isolated features. They do not establish multi-dimensional correlations with auxiliary Whois information (e.g. prefixes indicating the administrator or country), resulting in migrated patterns that struggle to fully align with the actual network structures of nonseed prefixes in some scenarios, thereby generating invalid target addresses.

Third, the inability to address missing Whois information. Whois data contains critical auxiliary information such as IPv6 prefix maintainers, network names, and geographic regions, serving as the core basis for understanding prefix semantics and structure. However, existing algorithms lack adequate mechanisms to supplement missing Whois information for nonseed prefixes, significantly reducing the reliability of similarity-based retrieval and pattern migration.

To address this, we propose 6HAN, which constructs a heterogeneous graph from IPv6 prefixes and their Whois attributes. It learns embedded representations of seed prefixes using a self-supervised graph attention network, then generates high-quality target addresses for nonseed prefixes via embedded-similarity retrieval and pattern migration. This offers a novel method for efficiently detecting IPv6 active addresses for nonseed prefixes.

The main contributions of this study are as follows:

- We pioneer the integration of Graph Neural Networks (GNNs) into IPv6 nonseed prefix target generation. 6HAN constructs a heterogeneous graph by leveraging IPv6 prefixes and their associated Whois information, and employs a heterogeneous graph attention network to model multi-modal correlations. This work provides a structured framework and a novel graph learning approach for fusing multi-source features related to IPv6 prefixes.
- We design a self-supervised heterogeneous graph attention network framework for model training. By generating prefix embeddings and performing dual-task collaborative optimization, the algorithm dynamically adjusts the contribution weights of auxiliary Whois information to the semantic representation of prefixes, fully leveraging its value.
- Leveraging the association prediction capabilities of graph neural networks, we achieve precise completion of core Whois fields of nonseed prefixes. We provide reliable attribute support for similarity search and pattern migration, fundamentally improving the reliability and efficiency of nonseed prefix detection.
- Experimental results demonstrate that when sending 50 million probe packets, compared to existing algorithms, 6HAN achieves a 13.81% to 201.05% improvement in IPv6 address hit rates and a 17.41% to 233.58% increase in IPv6 prefix coverage. Furthermore, ablation experiments are designed to validate the effectiveness of the Whois information completion strategy.

The open-source code for 6HAN is available at: https://github.com/flyzhouyx/6HAN.

The subsequent structure of this paper is as follows: Section "Related works" reviews the research foundations related to IPv6 target generation and graph representation learning. Section "Design of 6HAN" details the methodological design of the 6HAN model, encompassing heterogeneous graph construction, training of the self-supervised heterogeneous graph attention network, nonseed prefix target address generation, and resolution of alias prefixes and alias addresses. Section "Experimental results and analysis" experimentally validates the performance advantages of 6HAN. Section "Conclusion" concludes the paper and outlines future research directions.

## Related works

To efficiently detect active IPv6 addresses, researchers have designed and implemented algorithms that generate IPv6 targets from both seed prefixess and nonseed prefixes. Notably, graph representation learning excels at associative modeling of multi-source heterogeneous data, demonstrating significant advantages across domains such as network analysis and feature fusion.

### IPv6 target generation algorithms for seed prefixes

IPv6 target generation algorithms for seed prefixes focus on address pattern mining and feature learning, incorporating technical methods, including clustering, reinforcement learning, and deep learning, to continuously improve the generation efficiency and hit rate of target addresses.

In 2016, Foremski et al.^11^ proposed Entropy/IP, which quantifies the variability of each nybble (half-byte) in IPv6 addresses using information entropy. They built a Bayesian network model based on segment-wise dependencies to enable address generation. In 2017, Murdock et al.^5^ proposed 6Gen, pioneering the use of the Agglomerative Hierarchical Clustering (AHC) algorithm for IPv6 address generation. In 2019, Liu et al.^7^ introduced 6Tree, which employs Division Hierarchical Clustering (DHC) and a leftmost dimension splitting strategy to partition the address space. In 2020, Song et al.^12^ proposed the DET algorithm, which partitions the address space by nybble entropy to aggregate high-activity dimensions. Meanwhile, Cui et al.^13^ introduced 6GCVAE, pioneering the application of deep learning in this field by leveraging a gated convolutional variational autoencoder to learn address structural features. Concurrently, Cui et al.^14^ proposed 6VecLM, which combines nybble and location indices as vector inputs to a Transformer model for target address generation.

In 2021, Hou et al.^4^ proposed 6Hit, integrating reinforcement learning into address scanning to adjust scanning directions dynamically. Cui et al.^15^ introduced 6GAN, which optimizes address generation via iterative adversarial training between a Generative Adversarial Network (GAN) and a reinforcement learning agent. In 2022, Yang et al.^16,17^ successively proposed 6Graph and 6Forest: the former performs density clustering by mapping addresses onto an undirected graph, while the latter prioritizes splitting dimensions with fewer outlier addresses to reduce interference. Song et al.^9^ introduced AddrMiner, which categorizes address space processing by seed count and combines reinforcement learning with organizational association strategies for pattern mining. In 2023, Hou et al.^3,8^ proposed 6Scan and HMap6: the former achieves asynchronous dynamic scanning via regional encoding, while the latter constructs a dual-address-space tree by fusing optimized AHC and DHC. Liu et al.^18^ introduced 6Former, treating bytes as tokens and learning address features via multi-head attention mechanisms and Transformer models.

In 2024, Williams et al.^6^ proposed 6Sense, leveraging weighted reinforcement learning and Long Short-Term Memory (LSTM) networks to generate prefixes and match address patterns. Song et al.^10^ enhanced AddrMiner by optimizing its reinforcement learning component via multi-minimum-entropy parallel partitioning and multi-armed bandit models. He et al.^19^ introduced 6Diffusion, which uses Bidirectional Encoder Representations from Transformers-based diffusion models for target generation. Zhang et al.^20^ proposed 6Vision, pioneering the application of image encoding techniques in IPv6 address generation. In 2025, Sun et al.^21^ proposed 6Loda, combining pattern filtering and ensemble learning to detect outliers and identify active addresses. Yang et al.^22^ proposed a diffusion-based detection model that classifies addresses by pattern, jointly trains Diffusion and Transformer models to generate addresses.

### IPv6 target generation algorithms for nonseed prefixes

The IPv6 addresses generated by existing IPv6 target generation algorithms are concentrated within IPv6 prefixes that include the seed addresses. To detect active IPv6 addresses in nonseed prefixes, researchers have developed dedicated IPv6 target generation algorithms for this scenario.

Hou et al.^8^ proposed HMap6, adopting a heuristic seed address collection algorithm to convert nonseed prefixes into seed prefixes. They also introduced an address pattern mining method that integrates hierarchical and split-based clustering. Specifically, HMap6 traverses all subnet prefixes of a specific length under nonseed prefixes, selecting either a low-byte address with an Interface Identifier (IID) set explicitly to "::1" or a random IPv6 address to perform scanning for each subnet prefix. HMap6 is restricted to these two address types, resulting in a low hit rate.

Song et al.^9^ proposed AddrMiner-N, which establishes associations between seed and nonseed prefixes using the organization field in Whois information, migrating seed address patterns to nonseed prefixes. Subsequently, Song et al.^10^ improved AddrMiner-N by incorporating the organization, country, and keyword fields as joint association factors for IPv6 prefixes.

### Graph representation learning

Heterogeneous graph representation learning is a core branch of graph representation learning, focusing on modeling complex structures involving multi-type nodes and edges. It embeds nodes into low-dimensional Euclidean spaces while preserving heterogeneous structural characteristics and deep semantic information, providing robust support for modeling associations in multi-source heterogeneous data. Notably, Graph Neural Networks have demonstrated strong capabilities in modeling complex relational data, particularly in mining correlations from multi-source heterogeneous information. The IPv6 prefixes and their Whois attributes naturally form a heterogeneous relational network: prefix nodes are linked to various entities, such as maintainers, countries, and keywords, through multiple semantic associations that are not only structural but also carry rich administrative semantics and business logic. Traditional methods, such as rule-based matching or shallow clustering, struggle to fully capture such multi-modal relationships, whereas GNNs can aggregate multi-hop neighborhood information through message-passing mechanisms, thereby learning more discriminative prefix representations. Specifically, IPv6 prefix datasets exhibit learnable characteristics in terms of structure, semantics, and transferability. Structurally, they contain explicit relational edges (e.g. "same maintainer" or "same network name" relations). Semantically, they carry rich Whois field signals (e.g. organization, country, keywords). These signals can be fused via node attribute embeddings. For transferability, patterns from seed prefixes are migratable (e.g. address-allocation habits, service-type preferences). The patterns can be transferred to nonseed prefixes using semantic similarity in the embedding space. These properties make GNNs a suitable and promising approach for IPv6 target generation.

Compared to homogeneous graph representation learning, heterogeneous graph representation learning faces key challenges, including multi-type entity attribute fusion and the capture of complex relational semantics, which have driven the development of several representative methods. Among these, Wang et al.^23^ proposed the Heterogeneous Graph Attention Network, which stands out via its innovative dual-layer attention mechanism. Its node-level attention assigns distinct importance to neighboring nodes for the same relationship type, while its semantic-level attention dynamically learns the contribution weights of different relationships. This enables adaptive extraction of core associations without manual intervention, demonstrating enhanced flexibility and discriminative capability in multi-modal data modeling.

Heterogeneous graph representation learning has achieved successful applications in cybersecurity and knowledge graphs^24–29^. For instance, in malicious domain detection, integrating heterogeneous entities, e.g. domains, IP (Internet Protocol) addresses, and Domain Name System (DNS) servers, markedly improves the accuracy of identifying malicious activity. In text mining and recommendation systems, cross-type associative modeling enhances semantic understanding and recommendation effectiveness. However, in the field of IPv6 address detection, the potential of heterogeneous graph representation learning remains largely unexplored. Existing studies have not yet applied it to fuse the topological structure of IPv6 prefixes and Whois semantic attributes, nor have they developed targeted designs for mining nonseed prefix patterns. This creates significant research gaps that the proposed 6HAN model in this paper aims to address.

## Preliminaries

To better understand the paper, we first introduce key symbols and concepts to standardize the problem description, then formulate the IPv6 nonseed prefix target generation problem in a rigorous mathematical framework, clarifying the input, output, core objectives, and constraints.

### Definition

The key symbols and their mathematical definitions are summarized below, providing a unified foundation for subsequent problem modeling and algorithm description:

Definition 1: Seed prefix set *S*. $S=\{s_1,s_2,...,s_M\}$, where $s_i$ denotes a seed prefix with complete Whois information and known active address patterns.

Definition 2: Nonseed prefix set *N*. $N=\{n_1,n_2,...,n_K\}$, where $n_j$ denotes a nonseed prefix with incomplete Whois information.

Definition 3: Whois information matrix *W*. $W=[W_S; W_N]$, where $W_S\in R^{M\times F}$ denotes complete seed Whois attributes, $W_N\in R^{K\times F}$ denotes incomplete nonseed Whois attributes (missing entries marked as $\emptyset$), and $F=5$ (core fields: Mnt, Netname, Country, Keyword, Status).

Definition 4: Heterogeneous graph *G*. $G=(V,E,T)$, $V=V_P\cup V_A$ ($V_P=S\cup N$ is prefix nodes, $V_A$ is auxiliary nodes), *E* is edge set (7 types), *T* maps nodes/edges to their types.

Definition 5: Seed address pattern library $P_S$. $P_S=\{p_{s_i}|s_i\in S\}$, where $p_{s_i}$ is the set of active address patterns mined from $s_i$.

Definition 6: Total probe budget *B*. Maximum number of probe packets allowed ($B>0$).

Definition 7: Generated target address set *A*. $A\subseteq \bigcup _{n_j\in N}Addr(n_j)$, where $Addr(n_j)$ is all possible addresses under $n_j$.

Definition 8: Number of active addresses detected from *A* *Hit*(*A*). $Hit(A)=\sum _{a\in A}I(a\in A_{active})$, where $A_{active}$ is the set of active addresses.

Definition 9: Number of nonseed prefixes covered by active addresses *Cov*(*A*). $Cov(A)=|\{n_j\in N\mid \exists a\in A\cap Addr(n_j),I(a\in A_{active})=1\}|$.

Definition 10: Completed nonseed Whois matrix $\hat{W}_N$. Supplemented from $W_N$.

Definition 11: True nonseed Whois matrix $W_N^*$. Ground truth for completion error evaluation.

### Problem statement

The core problem in IPv6 nonseed prefix target generation is to generate high-quality target addresses under nonseed prefixes with limited probe budgets, addressing the challenges posed by incomplete Whois information and the lack of seed patterns.

The input is shown as Eq. (1):

1 $$\begin{aligned} I=(S,N,W,B), \end{aligned}$$

where $W_S$ has no missing entries (i.e. $W_S[i,f]\ne \emptyset \text { for all }i\in [1,M],f\in [1,F]$), $W_N$ contains missing entries (i.e. $\exists j\in [1,M],f\in [1,F]$ such that $W_N[j,f]=\emptyset$), and *B* is a predefined computational or storage constraint.

The output is a target address set $O=A$, which must satisfy $|A|\le B$ (budget constraint) and ensure all $a\in A$ are derived from migrated seed patterns (pattern validity constraint).

This problem is a prioritized multi-objective optimization task aiming to balance detection efficiency, comprehensiveness, and reliability.

The primary objective is to maximize the address hit rate, which reflects the proportion of active addresses detected per probe packet and is formalized as Eq. (2):

2 $$\begin{aligned} \max _{A,|A|\le B}HR(A)=\frac{Hit(A)}{|A|}. \end{aligned}$$

The second objective is to maximize prefix coverage, which quantifies the number of nonseed prefixes with detected active addresses and is defined as Eq. (3):

3 $$\begin{aligned} \max _{A,|A|\le B}PC(A)=|\{n_j\in N\mid \exists a\in A\cap Addr(n_j),I(a\in A_{active})=1\}|. \end{aligned}$$

The third objective is to minimize Whois information completion error to support reliable pattern migration, expressed as Eq. (4):

4 $$\begin{aligned} \min _{\hat{W}_N}CE(\hat{W}_N)=\frac{1}{K\times F}\sum _{j=1}^K\sum _{f=1}^FI(\hat{W}_N[j,f]\ne W_N^*(j,f)).\end{aligned}$$

The optimization process is subject to two non-negotiable constraints:

- Pattern Validity Constraint: For any $a\in A$, there exists $s_i\in S,p_{s_i}\in P_{S,}$, and $n_j\in N$ such that $a=Migrate(p_{s_i},n_j)$, where $Migrate(\cdot )$ replaces the fixed segment of $p_{s_i}$ with the fixed segment of $n_j$.
- Budget Constraint: The total number of generated addresses cannot exceed the predefined probe budget, i.e. $|A|\le B$.

## Design of 6HAN

**Fig. 1:**
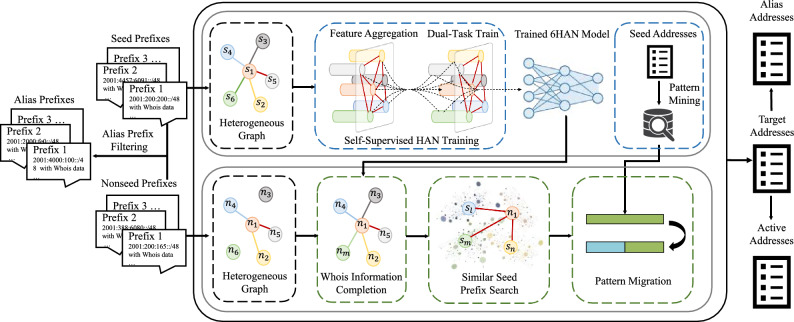
Workflow of 6HAN.

This section begins with an overview of 6HAN, followed by a detailed explanation of its technical principles (Fig. 1).

### 6HAN overview

6HAN learns semantic and structural features of IPv6 seed prefixes via a heterogeneous graph attention network, generating viable target addresses for nonseed prefixes. The model consists of four core modules: heterogeneous graph construction, self-supervised training, nonseed prefix target generation, and alias resolution.

The heterogeneous graph construction module integrates Whois attribute data and active address detection logs to extract six specific entity types (e.g. Prefix, Mnt, Netname). Prefix entities adopt nybble encoding and prefix-length normalization as core features, forming edges to construct feature vectors using management-scale and term-frequency metrics. These entities act as nodes, with relationships between them forming edges, creating a structured graph. The self-supervised training module adopts an enhanced HAN architecture: node-level attention mechanisms learn the contribution weights of distinct relationship types, generating entity embeddings that encapsulate both semantic and structural properties. Meanwhile, the model is optimized via joint minimization of a contrastive loss (based on hierarchical positive constraints for maintainers, countries, and keywords) and a link prediction loss (which predicts associations between prefixes and auxiliary entities).

In the nonseed prefix target generation module, the system first predicts and supplements missing fields in the Whois information of nonseed prefixes. It then retrieves similar seed prefixes and performs address pattern migration, balancing detection efficiency and cost via total budget control to output high-quality candidate addresses. Additionally, it filters out alias prefixes from seed and nonseed prefixes, and alias addresses from active target addresses, leveraging the IPv6 Hitlist alias database—thereby minimizing resource waste during the detection process.

The subsequent sections elaborate on each of these four modules in detail.

### Heterogeneous graph construction

For the unstructured description field in Whois information, 6HAN first uses the Natural Language Toolkit (NLTK) tokenizer to segment text, removes stop words while preserving valid terms, then computes term weights via Term Frequency-Inverse Document Frequency (TF-IDF), and finally extracts core keywords (e.g. extracting "Sony" and "Laboratories" from "Sony Computer Science Laboratories").

Meanwhile, we identify heterogeneous graphs as the optimal solution. Their core advantage lies in their ability to model multi-type nodes and edges, which inherently aligns with the multi-modal associative properties of IPv6 prefixes. Furthermore, the subsequent attention mechanism enables dynamic learning of relationship weights without manual intervention. Thus, we construct a heterogeneous graph to convert raw Whois data into high-quality structured inputs suitable for graph attention-based learning.

**Algorithm 1:**
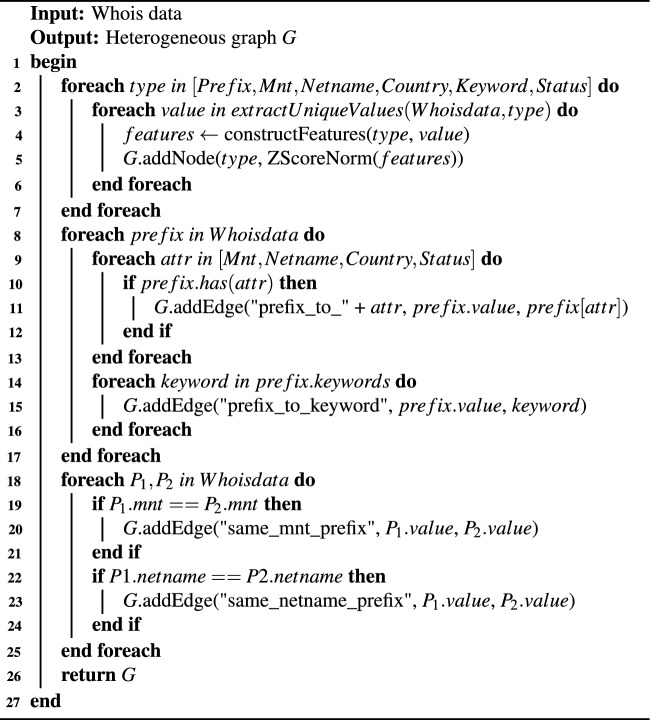
Heterogeneous graph construction.

During heterogeneous graph construction, 6HAN first semantically defines nodes and edge types. Specifically, by drawing on the core attribute dimensions of IPv6 prefixes, it defines six node types and seven edge types, as shown in Tables 1 and 2, respectively.

**Table 1:** Heterogeneous graph node definition.

Node type	Semantic function	Data source	Example
Prefix	Core entity	inet6num field	2001:200:132::/48
Mnt	Administrative entity identifier	mnt-by field	MAINT-JP-WIDE
Netname	Business network identifier	netname field	SONYCSL-NET
Country	Geographic attribute identifier	country field	JP
Keyword	Business semantic identifier	descr field extraction	SONY, COMPUTER
Status	Permission identifier	status field	ASSIGNED NON-PORTABLE

**Table 2:** Heterogeneous graph edge definition.

Edge type	Head node	Tail node	Semantic function
Prefix to Mnt	Prefix	Mnt	Prefix association with its maintainer
Prefix to Netname	Prefix	Netname	Prefix associated with its network name
Prefix to Country	Prefix	Country	Prefix associated with its country of origin
Prefix to Keyword	Prefix	Keyword	Prefix associated with its business keywords
Prefix to Status	Prefix	Status	Prefix associated with its allocation status
Same Mnt Prefix	Prefix	Prefix	Association between prefixes belonging to the same maintainer
Same Netname Prefix	Prefix	Prefix	Association between prefixes belonging to the same network name

**Table 3:** Heterogeneous graph node features.

Node type	Feature dimension	Feature construction method
Prefix	33	1. IPv6 Address Nybble Encoding (32-dimensional): Expand the IPv6 address into 32 nybbles, with each nybble mapped to a hexadecimal number. 2. Prefix Length Normalization (1-dimensional): Divide the prefix length by 128 (the maximum IPv6 prefix length), mapping it to the [0, 1] interval.
Mnt	2	1. Maintenance Scale Normalization (1-dimensional): The ratio of a maintainer’s prefix count to the maximum prefix count across all maintainers. 2. Regional Identifier (1-dimensional): 1 if it contains a country code, otherwise 0.5.
Netname	2	1. Prefix Proportion (1-dimensional): The length of the network name divided by the maximum length of all Netnames. 2. NET Identifier (1-dimensional): 1 if it contains the NET substring, 0 otherwise.
Country	2	1. The number of prefixes for the country is divided by the total number of prefixes. 2. Regional Classification (1-dimensional): The proportion of total prefixes that belong to the country’s geographic region (e.g. Asia, North America).
Keyword	2	1. Term Frequency Normalization (1-dimensional): The frequency of a keyword appearing in the descr field of all prefixes divided by the maximum term frequency. 2. Organization Association (1-dimensional): The proportion of times a keyword co-occurs with the same Mnt relative to the total occurrence count of that keyword.
Status	2	1. Portability Indicator (1-dimensional): PORTABLE is 1, otherwise 0. 2. Assignment Status Indicator (1-dimensional): ASSIGNED is 1, otherwise 0.

Subsequently, the node features of the heterogeneous graph must effectively encode the semantic information of the respective entities. To this end, we adopt distinct feature construction methods tailored to the unique attributes of the six entity types, ensuring consistent feature dimensions. Table 3 presents the feature dimensions and corresponding construction methods for each entity type. All entity features are subjected to Z-score normalization (with a mean of 0 and a standard deviation of 1) using the following formula:

5 $$\begin{aligned} x_{normalization} = \frac{x - \mu }{\sigma }, \end{aligned}$$

where ${\mu }$ is the mean of the feature, ${\sigma }$ is the standard deviation of the feature, to avoid the impact of feature magnitude differences on subsequent attention weight calculations.

Finally, 6HAN initializes all nodes and constructs edges in accordance with the semantic specifications of the seven defined relationship types. Specifically, if the attributes of two entities satisfy the relationship criteria summarized in Table 2, an edge is instantiated between them. During this procedure, edges are created for all qualifying entity pairs, with both the relationship type and its direction explicitly annotated. Each edge is subsequently associated with the node identifiers of the corresponding entities, thereby ensuring the traceability and interpretability of inter-entity relationships.

It is important to note that two specific relationship types—Same Mnt Prefix and Same Netname Prefix—correspond to Prefix-to-Prefix associations. Their construction rules are defined as follows: if two prefixes share the same maintainer (mnt-by) attribute or network name (Netname) attribute, a directed edge is added between them to enable subsequent similarity learning tasks. The pseudocode detailing the heterogeneous graph construction process is presented in Algorithm 1.

### Self-supervised heterogeneous graph attention network training

The core goal of the training phase is to learn high-quality low-dimensional vector embeddings for IPv6 prefixes, ensuring that prefixes with similar semantic and structural properties (e.g. those sharing the same maintainer or service type) are positioned closer in the embedding space while accurately capturing the associative relationships among entities.

#### Heterogeneous graph attention feature aggregation

**Fig. 2:**
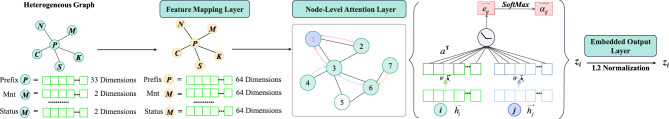
Heterogeneous graph attention feature aggregation.

The heterogeneous graph attention feature aggregation module comprises three components: the feature mapping layer, the node-level attention layer, and the embedding output layer. This module ultimately produces a prefix node embedding with strong discriminative power. Its overall structure is depicted in Fig. 2, and the pseudocode for heterogeneous graph attention-based feature aggregation is presented in Algorithm 2.

**Algorithm 2:**
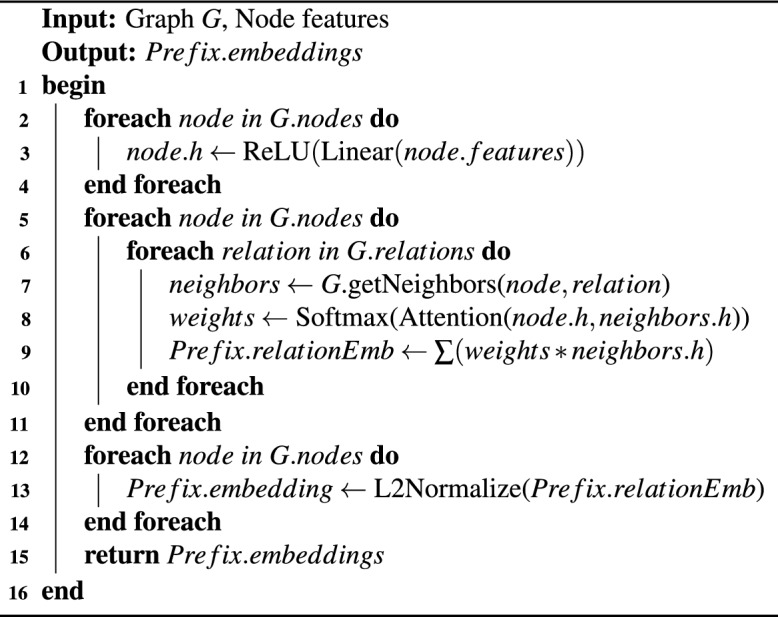
Graph attention feature aggregation.

The core objective of the feature-mapping layer is to eliminate discrepancies in feature dimensions and distributions across different entity types, laying a consistent foundation for feature input in subsequent attention computations. Its design is motivated by the significant heterogeneity of node features in heterogeneous graphs: for example, prefix nodes have 33-dimensional features, while auxiliary entities (e.g. Mnt and Netname) have only 2 dimensions. Using raw features directly would introduce biases into attention calculations, requiring all node features to be mapped to a unified dimension.

Within the feature mapping layer, 6HAN adopts independent linear mapping layers for each node type, transforming input features into 64-dimensional intermediate features in the hidden layer. The specific mapping process is as follows: for a prefix node *i*, intermediate features are obtained through the linear transformation shown in Eq. (6).

6 $$\begin{aligned} h_{_t}=\textrm{ReLU}(W_{_p}\cdot x_{_t}+b_{_p}), \end{aligned}$$

where $x_i$ denotes the 33-dimensional feature vector of the prefix node constructed in Sect. "Heterogeneous graph construction", $W_{p}\in \mathbb {R}^{64\times 33}$ represents the weight matrix of the linear transformation for the prefix node, and $b_{p}\in \mathbb {R}^{64}$ denotes the corresponding bias term. For the remaining auxiliary nodes, taking the Mnt node $m$ as an illustrative example, its 2-dimensional raw feature vector $x_j$ is passed through the corresponding linear layer (see Eq. (7)) to obtain the intermediate feature representation.

7 $$\begin{aligned} h_{_j}=\textrm{ReLU}(W_{_m}\cdot x_{_j}+b_{_m}), \end{aligned}$$

where $W_{m}\in \textbf{R}^{64\times 2}$ represents the weight matrix for the linear mapping of the Mnt node, and $b_{m}\in \textbf{R}^{64}$ denotes the bias term. All mapped intermediate features are processed via the ReLU activation function, introducing nonlinearity to improve feature expressiveness and mitigate information loss induced by linear transformations.

After unifying feature dimensions, it is necessary to optimize further the contribution weights of auxiliary nodes to the prefix embedding of core nodes. To this end, 6HAN independently computes neighborhood attention for each edge category, divided into three steps:

Step 1 is attention score calculation. For any edge (*i*, *j*) (e.g. Prefix *i* pointing to Mnt *j*), the attention score $e_{ij}$ is calculated as follows:

8 $$\begin{aligned} e_{ij}=\textrm{LeakyReLU}(a^{\textrm{T}}\cdot [W\cdot h_{i}\Vert W\cdot h_{j}]), \end{aligned}$$

where *W* denotes a 64$\times$64 shared weight matrix (used to learn interaction information among entity features), *h* is a 128$\times$1 attention vector (used to learn the importance of features after concatenation), and "||" represents the vector concatenation operation.

We deliberately removed the semantic-level attention layer from the traditional HAN framework. This decision stems from the inherent "one-to-one" relationship between an IPv6 prefix and its auxiliary entities (e.g. Mnt, Country). In this context, the quadratic distribution of semantic-level attention offers no practical benefit. Moreover, our node-level attention layer already performs the necessary weighted fusion of multi-relationship features, making an additional aggregation layer redundant. This optimization reduces the attention complexity from $\textrm{O}(N\times R^2)$ to $\textrm{O}(N\times R)$ (where *N* is the number of nodes and *R* is the number of relationships).

To empirically validate this design choice, we conducted a ablation experiment between the full HAN architecture (with semantic-level attention, denoted as 6HAN-w/sem) and our streamlined variant (6HAN).

Step 2 involves attention normalization. All neighborhood attention scores for Prefix node *i* are normalized using the Softmax function to obtain the attention weight $\alpha _{ij}$ of neighborhood node *j* toward *i*. The calculation process is as follows:

9 $$\begin{aligned} \alpha _{ij}=\frac{\exp (e_{ij})}{\sum _{j^{\prime }\in N_{i}}\exp (e_{ij^{\prime }})}, \end{aligned}$$

where $N_{i}$ represents the neighborhood set of node *i*.

Step 3 is weighted fusion, which sums the intermediate features for each relationship type, weighted by their corresponding weights, to obtain the embedding vector $z_i$ for the prefix node. The calculation method is as follows:

10 $$\begin{aligned} z_i=\sum _{j\in N_i}\alpha _{ij}\times h_j. \end{aligned}$$

The core objective of the embedding output layer is to improve the standardization and discriminative capability of embedding vectors, ensuring the stability and accuracy of subsequent similarity calculations.

6HAN performs L2 normalization on the embedding vector $z_i$ output by the node-level attention layer, which is calculated as follows:

11 $$\begin{aligned} z_i=\frac{z_i}{\left\| z_i\right\| _2}, \end{aligned}$$

where ${\left\| z_i\right\| _2}$ denotes the Euclidean norm of vector $z_i$.

#### Dual-task joint self-supervised training

**Algorithm 3:**
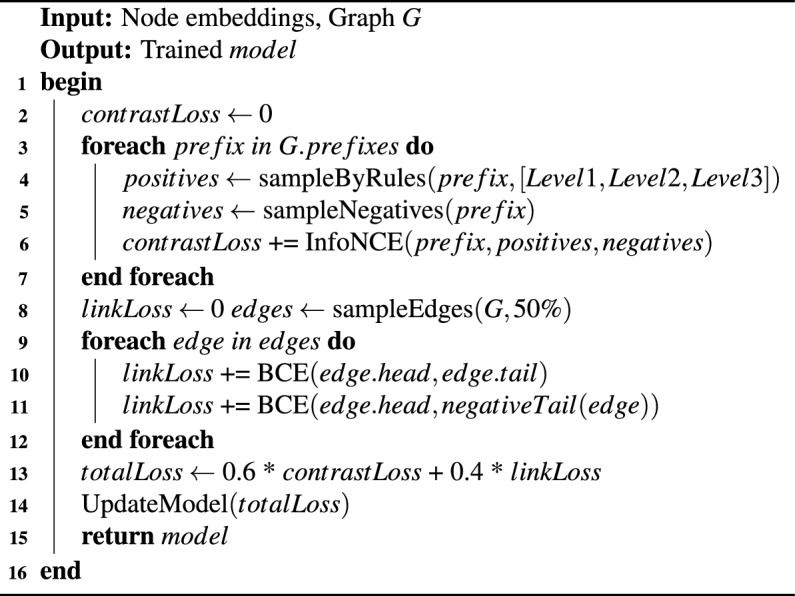
Dual-task joint self-supervised learning.

**Fig. 3:**
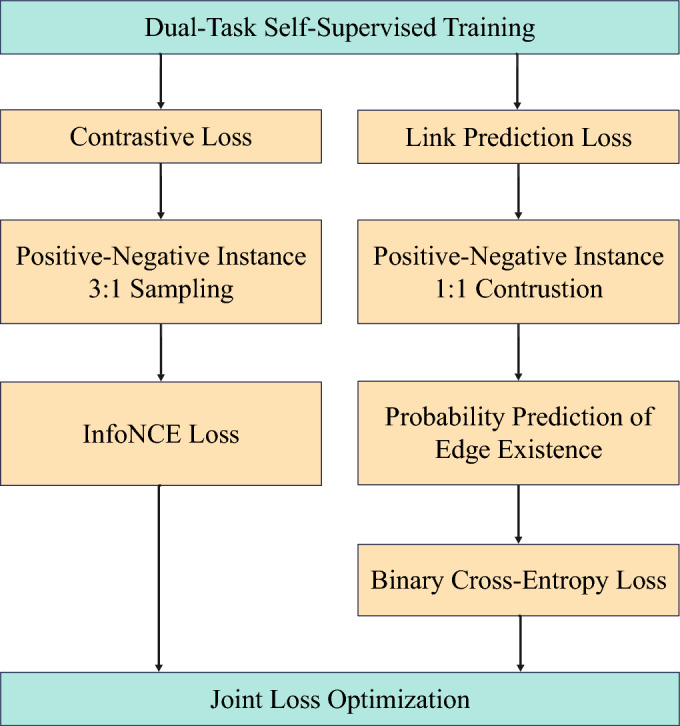
Workflow of dual-task joint self-supervised training.

To address the core challenge of unlabeled IPv6 prefix data, 6HAN designs a joint self-supervised training task that combines a contrastive loss with a link-prediction loss. The training process is illustrated in Fig. 3, and the pseudocode for dual-task joint self-supervised training is presented in Algorithm 3. In the contrastive loss task, the objective is to optimize the distribution of embedding distances in the latent space, such that semantically similar prefixes are mapped to nearby locations. To ensure the semantic reliability of the supervision signals, 6HAN constructs three levels of positive samples with progressively decreasing similarity, along with a single level of negative samples, as defined in Table 4.

**Table 4:** Positive and negative instance definitions for contrastive loss.

Type	Rule construction	Similarity	Sampling ratio
Level 1 positive instance	Prefixes *u* and *v* share the same maintainer and belong to the same business network	Highest similarity	30%
Level 2 positive instance	Prefixes *u* and *v* share the same maintainer but do not belong to the same business network	High similarity	40%
Level 3 positive instance	Prefixes *u* and *v* belong to the same country	Moderate similarity	20%
Negative instance	Does not satisfy the above positive instance rules	No similarity	10%

To balance the sample distribution during training, we adopt a sampling strategy that randomly samples three positive samples (one from each level: Level 1, Level 2, and Level 3) and one negative sample for each Prefix *u*, resulting in four (*u*, *v*) sample pairs. This ensures a balanced 3:1 positive-to-negative ratio, mitigating model bias induced by sample imbalance.

For the contrastive loss function, we selected InfoNCE, calculated as follows:

12 $$\begin{aligned} L_{contrast}=-\frac{1}{N}\sum _{i=1}^{N}\log \left( \frac{\exp (s(z_{i},z_{i^{+}}))}{\exp (s(z_{i},z_{i^{+}}))+\sum _{j\in N_{i}}\exp (s(z_{i},z_{j}))}\right), \end{aligned}$$

where *N* represents the total number of training samples, $z_i$ denotes the embedding vector for Prefix *i*, and $z_{i^{+}}$ is the set of positive examples for Prefix *i*. $N_i$ represents the set of negative examples for Prefix *i*. *s*(*a*, *b*) indicates the inner product of embedding vectors (since embeddings have undergone L2 normalization, the inner product result is equivalent to cosine similarity), used to quantify similarity between embeddings.

The core objective of the link prediction loss task is to enable embeddings to accurately capture and predict the existence of edges between prefix-auxiliary entities in the heterogeneous graph.

For the link prediction loss task, positive and negative samples are constructed using a sampling strategy. Positive samples are obtained by randomly selecting 50% of the genuine edges in the heterogeneous graph (e.g. (*u*, *v*) is a genuine edge). In contrast, the remaining true edges are reserved for the validation set.

Negative examples are generated by applying an entity replacement strategy to each positive example (*u*, *v*): the tail entity *v* is replaced with another entity of the same type (e.g. replacing *v* with $v'$ of the same type) such that there is no existing edge between *u* and $v'$.

The link prediction loss adopts binary cross-entropy (BCE) loss, computed in two steps:

First, probability prediction: the dot product of the two feature vectors is mapped to the interval [0, 1] using the Sigmoid function, yielding the predicted probability of edge existence:

13 $$\begin{aligned} \hat{y}=\sigma (h_u\cdot h_v), \end{aligned}$$

where $\sigma (\cdot )$ represents the sigmoid function, $\hat{y}=1$ indicates the predicted edge exists, and $\hat{y}=0$ indicates the predicted edge does not exist.

Step 2 involves calculating the BCE loss, which quantifies the deviation between the predicted probability and the actual label using a logarithmic loss function. The calculation is as follows:

14 $$\begin{aligned} L_{link}=-\frac{1}{M}\sum _{k=1}^M\left[ y_k\log (\hat{y}_k)+(1-y_k)\log (1-\hat{y}_k)\right], \end{aligned}$$

where *M* denotes the total number of edge samples, and $y_k$ represents the true labels of edges (where ${ y_k}=1$ corresponds to positive instances and ${y_k}=0$ to negative instances).

**Fig. 4:**
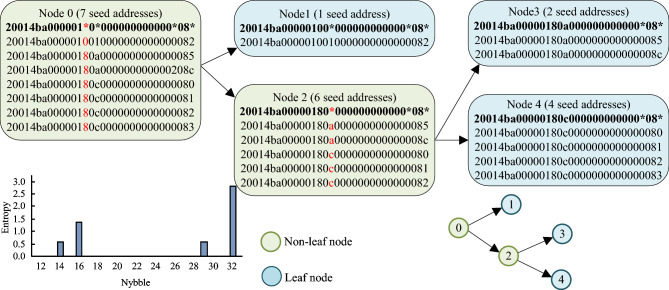
IPv6 Seed Address Clustering and Pattern Mining Using a Minimum Entropy Partitioning Strategy.

To balance the priorities of similarity learning and structural association learning, the contrastive loss and link prediction loss are linearly combined with weights to construct a joint loss function:

15 $$\begin{aligned} L_{total}=0.6\times L_{contrast}+0.4\times L_{link}. \end{aligned}$$

The weight coefficient $\alpha$ was selected via grid search on test sets.

### Nonseed prefix target generation

Based on the pre-trained model and seed prefix embeddings, 6HAN first generates embeddings for nonseed IPv6 prefixes, then performs link prediction on these nonseed prefixes to complete missing fields in their Whois information. Finally, it efficiently identifies semantically similar seed prefixes for each nonseed IPv6 prefix and migrates the address patterns associated with seed prefixes to the nonseed ones.

#### Nonseed prefix whois information completion

**Algorithm 4:**
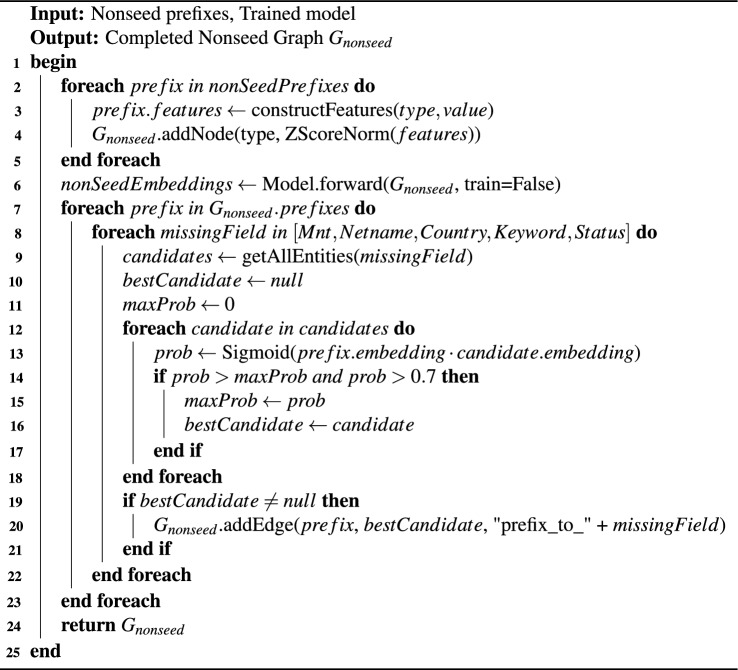
Nonseed prefix whois information completion.

The optimization objective of link prediction loss endows the model with the ability to learn the structural patterns governing the formation of edges between IPv6 prefix nodes and auxiliary attribute entities. In essence, the task of imputing missing Whois-related edges for nonseed prefixes can be formalized as a problem of predicting the existence of associative edges between nonseed prefixes and their corresponding missing attribute entities, whose optimization objective is fully aligned with the mathematical formulation of link prediction. Therefore, the pre-trained model and the node embedding representations learned by the model can be directly utilized to perform this Whois information completion task.

6HAN first preprocesses nonseed IPv6 prefixes, reusing the feature engineering method applied to seed prefixes to extract and preprocess features for each entity associated with each nonseed prefix. This guarantees complete consistency in feature representations between nonseed and seed prefixes. Subsequently, 6HAN constructs a graph over the nonseed prefixes, comprising the nonseed prefix entities themselves and their related entities (e.g. Mnt, Netname, Keyword) obtained from the Whois records of the nonseed prefixes. The feature definitions for all entities follow those specified in Table 3. In parallel, any missing entity information for a given nonseed prefix is explicitly flagged as pending completion.

Subsequently, the model trained on seed prefixes is used to generate embedding vectors for nonseed prefix nodes in the heterogeneous nonseed prefix graph. Specifically, 6HAN takes as input the temporarily constructed heterogeneous graph composed of nonseed prefixes. It performs forward propagation through the pre-trained model to obtain 64-dimensional embedding vectors for all nonseed prefix nodes, followed by L2 normalization of these vectors.

During this process, 6HAN keeps all learnable parameters fixed, including those of the feature mapping layer and the attention layer. In other words, 6HAN executes only a forward propagation step without any parameter fine-tuning, thereby avoiding disruption of the spatial distribution of the embeddings learned from seed prefixes.

Finally, performs a field requiring completion. 6HAN calculates the association probability between each nonseed prefix and candidate entities using Equation (13) from the link prediction loss training. The candidate entity with the highest probability exceeding the threshold is selected as the completion result. If the probabilities of all candidate entities are below $\theta$, no completion is performed to avoid erroneous filling. To determine the completion threshold, we conducted a sensitivity analysis on validation set by varying $\theta \in \{0.5,0.6,0.7,0.8,0.9\}$ and evaluating both address hit rate, Whois completion accuracy and coverage.

Consistency checks are then conducted: if the supplemented entity has a significant conflict with the existing information of the nonseed prefix (e.g. the supplemented Mnt is MAINT-JP-WIDE while the country in the nonseed prefix’s Whois information is US), the supplemented result is discarded. The pseudocode for nonseed prefix Whois information completion is presented in Algorithm 4. After completing the missing fields in the Whois information, 6HAN reconstructs the completed nodes and calculates their features. Based on the addition rules, it adds edges to the completed entities and updates the nonseed prefix heterogeneous graph.

#### Pattern migration and target generation

After completing the Whois information for nonseed prefixes, the heterogeneous graph of nonseed prefixes was updated. 6HAN fed it into a model trained on seed prefixes and their Whois information, generating node embedding vectors for nonseed prefixes and retrieving similar seed prefixes for pattern migration.

To achieve pattern migration, IPv6 addresses under seed prefixes must first undergo pattern mining to establish a high-quality seed address pattern library. Seed prefixes are matched with active IPv6 addresses in the Hitlist via longest prefix match to filter out a set of valid active addresses. The method then applies a minimum entropy segmentation strategy to perform hierarchical clustering on the addresses under each seed prefix, replacing variable nybbles with wildcards. "*" to form the pattern library (as shown in Fig. 4).

**Fig. 5:**
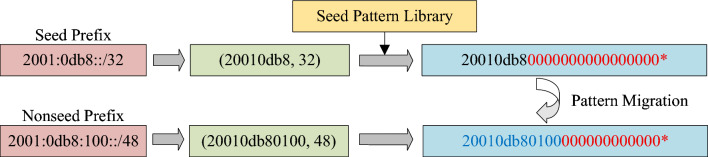
Pattern migration for nonseed prefix.

During pattern migration, the first *L* nybbles of the seed pattern are replaced with the fixed prefix segment of the nonseed prefix, while the remaining $(32-L)$ nybbles are retained. This generates a valid target address pattern for the nonseed prefix. As shown in Fig. 5, for a seed pattern 20010db80000000000000000* (derived from a /32 seed prefix, where $L=8$ nybbles correspond to the fixed /32 segment 20010db8), migrating it to a /48 nonseed prefix with the fixed segment 20010db80100 (where $L=12$ nybbles) yields the nonseed pattern 20010db80100000000000000*. To balance scan coverage with computational and storage costs, 6HAN first sets an overall generation budget and distributes it evenly among all valid nonseed prefixes to determine the per-prefix generation quota. Simultaneously, to prevent any single migration pattern from consuming excessive resources and undermining multi-pattern coverage diversity, a maximum generation limit is established for each individual migrated pattern.

In the target address generation phase, for each migration mode associated with a nonseed prefix, the number of generated addresses does not exceed the per-mode maximum limit until the prefix’s individual quota is exhausted. This process ultimately yields a set of IPv6 target addresses ready for direct scanning.

### Alias prefix and alias address resolution

Alias addresses (i.e. multiple addresses used by the same node or network interface) and alias prefixes (where all addresses under the prefix are alias addresses) can introduce biases into the IPv6 target addresses generated by the algorithm. This means that most target addresses may belong to the same network interface, leading to unnecessary waste of detection resources^30^.

To mitigate the adverse impact of alias prefixes and addresses on detection accuracy and resource efficiency, 6HAN filters alias prefixes on both seed and nonseed prefixes. It applies alias address filtering to active addresses among the generated nonseed target addresses.

First, alias prefixes are removed from both seed and nonseed prefixes. Before data preprocessing and heterogeneous graph construction, these prefixes are matched against the IPv6 Hitlist alias prefix list to identify and fix resources.

Second, alias addresses are removed from active target addresses. For the detected active addresses among the generated nonseed target addresses, longest prefix matching is performed again. It applies the alias prefix list mentioned in the IPv6 Hitlist to eliminate alias addresses.

## Experimental results and analysis

This section provides a detailed description of the experimental setup and the methods used to construct the training and test sets. It compares the performance of 6HAN with non-seed-prefixed IPv6 address target generation algorithms (HMap6 and AddrMiner-N) on the IPv6 Hitlist and presents the results of ablation experiments.

### Experimental setup

The experiment was conducted in a real network environment on a cloud server equipped with 8GB of memory, an Intel Xeon Platinum processor (2.5GHz), and the Ubuntu 20.04 operating system. The experiment was completed in October 2025.

For 6HAN, the number of training iterations is set to 100, and the hierarchical clustering node-splitting threshold is set to 3. HMap6 operates in two modes: HMap6-low and HMap6-rand. It verifies for the experiments. For AddrMiner-N, the number of threads is configured as 30, and the number of detection rounds is set to 12.

We adopt the following metrics to evaluate the performance of IPv6 target generation algorithms:

*(1) IPv6 address hit rate: *The ratio of detected IPv6 addresses to the number of probe packets sent.

*(2) IPv6 prefix coverage: *The number of Border Gateway Protocol (BGP) prefixes covered by the detected IPv6 addresses.

To ensure statistical soundness of the experimental results, we report 95% confidence intervals (CIs) for all core metrics. The confidence intervals are calculated as follows:

*(1) For hit rate:* Adopt the Wilson score interval (suitable for binomial distributions) to address potential bias from small sample sizes, computed as $\hat{p}\pm z_{\alpha /2}\sqrt{\frac{\hat{p}(1-\hat{p})+z_{\alpha /2}^2/(4n)}{n+z_{\alpha /2}^2}}$, where $\hat{p}=\textrm{Hit}(A)/|A|$ (observed hit rate), $n=\left| A\right|$ (number of probe packets), and $z_{\alpha /2}=1.96$ (95% confidence level critical value).

*(2) For prefix coverage:* Use the Poisson confidence interval (based on the normal approximation for large counts), computed as $X\pm z_{\alpha /2}\sqrt{X}$, where *X* denotes the observed count (prefix coverage or active address number).

Given the extremely large number of probe packets (up to 50 million) and the correspondingly large number of detected active addresses, the 95% confidence intervals for hit rate are inherently narrow (typically ±0.02% to ±0.05% relative). This is an expected consequence of the binomial proportion’s standard error scaling as $\sqrt{\hat{p}(1-\hat{p})/n}$, where *n* is the number of probe packets. Consequently, even small absolute differences in hit rate (e.g. 0.1%) may yield non-overlapping confidence intervals and achieve statistical significance at $p<0.05$. While we report confidence intervals for completeness, the practical significance of our results is better assessed by the relative improvement percentages (e.g. 13.81% to 201.05% over baselines) and the absolute number of additional active addresses detected, which directly reflect the algorithm’s value in real-world scanning scenarios.

To help readers assess the deployability of 6HAN, we report the peak memory usage measured during training and inference on our test platform (8GB RAM, Ubuntu 20.04). During training with 100 epochs and a batch size of 64, the model consumes a peak of 3.2 GB of memory, primarily due to storing intermediate node embeddings, attention weights, and the heterogeneous graph structure (approximately 200k prefix nodes, 800k auxiliary nodes, and 2.4M edges). For inference on nonseed prefixes (forward propagation without gradient computation), the peak memory usage reduces to 1.5 GB. These figures indicate that 6HAN can be deployed on standard commodity servers without requiring specialized hardware.

### Construction of IPv6 prefix dataset

#### Training dataset construction

The training dataset required for 6HAN consists of seed prefixes and their corresponding Whois information. To enhance the model’s training quality, seed prefixes should be sourced from diverse origins, and Whois information should be as complete as possible. To this end, we acquired seed prefixes and their Whois information from multiple data sources, as shown in Table 5.

**Table 5:** Data sources for training and testing sets.

Train data source	Quantity	Test data source	Quantity
APNIC	418791	RouteViews	269327
ZoomEye	439843	RIPE	218615
FOFA	158751	IPv6 Hitlist	199330
Total	1017385	Total	687272

The data sources we utilized comprise the following three:

- *Asia-Pacific Network Information Centre (APNIC)*^31^: IPv6 prefixes and Whois information collected by the APNIC, encompassing 418,791 entries of IPv6 prefixes and their corresponding Whois data.
- *ZoomEye*^32^: IPv6 prefixes and Whois information collected by the ZoomEye cyberspace search engine, comprising 439,843 entries.
- *FOFA*^33^: IPv6 prefixes and Whois information data collected by the FOFA cyberspace search engine, comprising 158,751 entries of IPv6 prefixes and their corresponding Whois information.

After merging and deduplicating the aforementioned IPv6 prefixes, 816,703 entries containing the prefixes and their Whois information were obtained. By performing longest prefix matching between the IPv6 Hitlist and all prefixes, we obtained 263,927 seed prefixes and 552,776 nonseed prefixes. From the seed prefixes, 200,000 entries of prefixes with relatively complete Whois information were selected to construct the training dataset *S*. We then set aside 10% of the training set as the validation set $V_a$.

To further assess the feasibility of applying graph neural networks to IPv6 prefix data, we conduct a statistical learnability analysis on the constructed training set. The key structural and semantic characteristics are summarized in Table 6, which confirms that the dataset exhibits clear relational patterns and sufficient completeness to support effective representation learning.

Statistical data indicate that IPv6 prefix datasets exhibit structural regularity (multiple nodes/relationship types, moderate connectivity), semantic richness (high Whois completeness, keyword concentration), and management clustering (prefixes grouped by maintainer). These characteristics collectively demonstrate the applicability of graph-based learning methods, as the data inherently contains learnable associative relationships suitable for modeling.

To further quantify learnability, we evaluate two additional metrics. All evaluations are completed after model training on seed prefixes. The first metric is Link Prediction Area Under the Curve (AUC). It reaches 0.882 on the held-out validation set. This result indicates that the heterogeneous graph structure encodes strong relational patterns. The model can effectively capture these patterns. The second metric is the intra/inter-class similarity ratio. We calculate this ratio in two steps. First, we get the average cosine similarity between embeddings of prefixes sharing the same maintainer. Second, we divide this value by the average cosine similarity between embeddings of prefixes with different maintainers. The final ratio is 2.34. This confirms that the learned embeddings cluster semantically related prefixes. They also separate dissimilar prefixes effectively. These metrics substantiate two key points. The training data has rich structural information. It also enables effective representation learning.

#### Test dataset construction

We collected IPv6 prefixes from public data sources. The selected data sources and their respective numbers of IPv6 prefixes are shown in Table 5.

**Table 6:** Learnability analysis of the training dataset.

Metric	Distribution	Implication for learnability
Node types	6 (e.g. Prefix, Mnt)	Provides the structural basis for a heterogeneous graph.
Edge types	7 (e.g. Prefix→Mnt)	Captures multiple semantic associations between prefixes and attributes.
Average node degree	4.2	Most prefixes are connected to several auxiliary entities, enabling neighborhood aggregation.
Whois primary field integrity	Maintainer: 82.1%, Country: 88.7%	High completeness supplies reliable supervised signals for training.
Prefixes per maintainer	3.8 / 412	Reflects administrative aggregation.
Keyword coverage	65.3% of prefixes	Shows concentrated business semantics, which facilitates keyword-aware similarity retrieval.
Link Prediction AUC (Validation Set)	0.882 ± 0.006	High AUC indicates that the graph structure encodes strong relational patterns that the model can effectively capture, validating the feasibility of using GNNs to predict prefix-auxiliary edges.
Intra/Inter-class Similarity Ratio	2.34 (intra-class cosine similarity: 0.72; inter-class: 0.31)	Ratio > 1 indicates that the learned embeddings cluster semantically related prefixes (same maintainer) while separating dissimilar ones, confirming the discriminative power of the representation learning.

The data sources we utilized include the following three:

- *RouteViews*^34^: BGP data from the University of Oregon’s RouteViews project, encompassing 269,327 IPv6 prefixes.
- *RIPE*^35^: BGP routing data collected by Réseaux IP Européens Network Coordination Centre, the European Internet registry, containing 218,615 IPv6 prefixes.
- *IPv6 Hitlist*^36^: Compiled by Gasser et al. from multiple sources, comprising non-alias active IPv6 addresses 22,506,867 and IPv6 prefixes 199,330 of varying lengths.

**Fig. 6:**
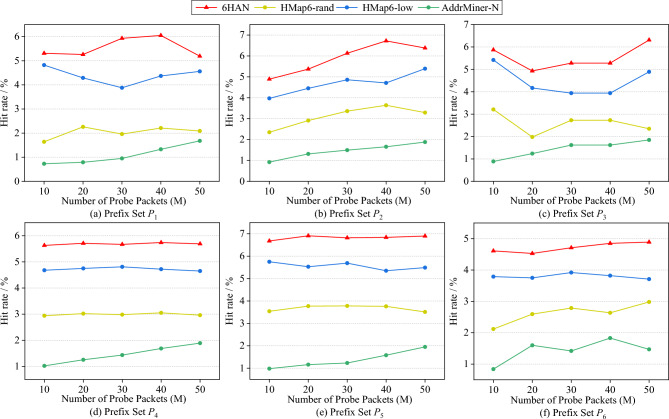
Comparison of IPv6 address hit rates.

After merging and deduplicating the aforementioned IPv6 prefixes, 594,921 prefixes were obtained. Longest prefix matching was performed between the IPv6 Hitlist and all prefixes to separate seed and nonseed prefixes. And we obtained 142,782 seed prefixes and 452,139 nonseed prefixes. Since the 6HAN model requires Whois information as input, but the aforementioned platforms did not provide Whois data corresponding to the prefixes, we adopted Whois command-based querying. Any fields that remained missing after scanning were marked as empty.

To thoroughly test and compare the performance of 6HAN and other algorithms, we constructed the test dataset using two approaches. First, the nonseed prefixes and their Whois information data were divided into three parts based on address sources: prefixes and their Whois information collected from RouteViews formed test dataset $P_1$; prefixes and their Whois information collected from RIPE formed test dataset $P_2$; and prefixes and their Whois information collected from Hitlist formed test dataset $P_3$.

Subsequently, we merged the prefixes and their Whois information from the three address sources. From this combined dataset, 200,000 prefixes and their corresponding Whois information were randomly sampled to construct test datasets $P_4$, $P_5$, and $P_6$.

### Comparative analysis of experimental results

In this section, we first carry out a sensitivity analysis to determine the optimal threshold for Whois information completion. On this basis, we conduct a systematic performance comparison between our proposed 6HAN and state-of-the-art baseline algorithms (HMap6 and AddrMiner-N), with IPv6 address hit rate and IPv6 prefix coverage as the core evaluation metrics. Finally, we design targeted ablation experiments to validate the effectiveness of the two key designs of our model, namely the Whois missing information completion strategy and the semantic-level attention removal strategy, and provide an in-depth analysis of the experimental results.

#### Sensitivity analysis of whois completion threshold

To evaluate the threshold $\theta$ of our Whois completion strategy, we varied the confidence threshold $\theta$ from 0.5 to 0.9 on validation set $V_a$ with 50M probe packets. As illustrated in Table 7, the address hit rate remains above 6.8% for $\theta \in [0.6,0.8]$, peaking at 6.91% when $\theta$ = 0.7. Completion accuracy (measured on the validation set $V_a$) increases monotonically with $\theta$, from 78.3% at $\theta$ = 0.5 to 94.2% at $\theta$ = 0.9, while completion coverage decreases from 92.1% to 53.4%. The selected threshold $\theta$ = 0.7 achieves a balanced trade-off (89.6% accuracy, 81.3% coverage).

**Table 7:** Sensitivity analysis of the whois completion threshold $\theta$ on validation set $V_a$ (50M probe packets).

$\theta$	Hit rate (%)	Completion accuracy (%)	Completion coverage (%)
0.5	6.72 ± 0.02	78.3	92.1
0.6	6.85 ± 0.02	84.7	87.5
0.7	6.91 ± 0.02	89.6	81.3
0.8	6.88 ± 0.02	92.8	70.2
0.9	6.75 ± 0.02	94.2	53.4

#### Selection of loss weight $\alpha$

We evaluated $\alpha \in \{0.3,0.4,0.5,0.6,0.7,0.8\}$ using the IPv6 address hit rate as the performance metric under a fixed probe budget of 50 million packets. As shown in Table 8, $\alpha$ = 0.6 consistently achieves the highest hit rate across all three datasets. Excessively high $\alpha \left( \ge 0.7\right)$ overemphasizes contrastive similarity, weakening structural association modeling; conversely, low $\alpha \left( \le 0.5\right)$ underutilizes semantic information, reducing pattern migration precision. Therefore, we set $\alpha$ = 0.6 in our final model.

**Table 8:** IPv6 address hit rates (%) for different loss weight $\alpha$ on test sets $P_4$, $P_5$ and $P_6$ (50M probe packets).

$\boldsymbol{\alpha }$	$\boldsymbol{P_4}$ hit rate (%)	$\boldsymbol{P_5}$ hit rate (%)	$\boldsymbol{P_6}$ hit rate (%)
0.4	5.54 ± 0.02	6.58 ± 0.02	4.19 ± 0.02
0.5	5.75 ± 0.02	6.79 ± 0.02	4.52 ± 0.02
**0.6**	**5.89 ± 0.02**	**6.91 ± 0.02**	**4.88 ± 0.02**
0.7	5.83 ± 0.02	6.85 ± 0.02	4.72 ± 0.02
0.8	5.61 ± 0.02	6.63 ± 0.02	4.59 ± 0.02

Significant values are in bold.

#### Comparison analysis of IPv6 address hit rate

Hit rate is the core metric for evaluating IPv6 address detection efficiency. It reflects the proportion of active IPv6 addresses detected after the algorithm sends probe packets, directly indicating the algorithm’s ability to obtain more active addresses with limited probe packet overhead.

Figure 6 presents the address hit rates of each algorithm under different probe packet volumes (with 95% confidence interval error bars), where subfigures $(a)-(f)$ correspond to the results of prefix sets $P_1$ to $P_6$, respectively. It can be observed that across the six prefix datasets, 6HAN outperforms HMap6 (including its two modes: HMap6-low and HMap6-rand) and AddrMiner-N in IPv6 address hit rates under all probe packet volumes. For completeness, we report 95% confidence intervals (Wilson score intervals) for hit rates. Due to the large sample size (n = 50M), these intervals are extremely narrow (typically ±0.02% to ±0.05% relative). Consequently, the observed differences yield non-overlapping CIs and achieve statistical significance at $p<0.05$.

However, the practical significance of 6HAN’s advantage is better reflected by the magnitude of improvement. Specifically, on the $P_5$ prefix set, 6HAN achieves a hit rate of 6.91%, compared to 5.49% for HMap6-low, 3.51% for HMap6-rand, and 1.95% for AddrMiner-N. When sending 50 million probe packets, this translates to relative improvements of 25.9% over HMap6-low, 96.9% over HMap6-rand, and 254.4% over AddrMiner-N. Across all six datasets, the hit rate improvement ranges from 13.81% to 201.05%. These substantial margins—far exceeding typical measurement noise—demonstrate that 6HAN delivers meaningful gains in detection efficiency for real-world IPv6 scanning tasks.

**Table 9:** Comparison results of probing rates of IPv6 target generation algorithms.

Seed address set	IPv6 target generation algorithm	Number of detected IPv6 active addresses	Time cost (s)	Probing rate (Number per sec)
$P_1$	6HAN	**2,595,857**	273,441	9.49
	HMap6-low	2,280,166	**9,082**	**251.06**
	HMap6-rand	1,045,983	9,520	109.87
	AddrMiner-N	841,312	327,359	2.57
$P_2$	6HAN	**3,155,821**	170,585	18.53
	HMap6-low	2,445,197	**9,293**	**263.12**
	HMap6-rand	1,175,643	9,807	119.86
	AddrMiner-N	925,704	189,698	4.88
$P_3$	6HAN	**3,190,628**	172,466	18.50
	HMap6-low	2,695,814	9,931	**271.46**
	HMap6-rand	1,645,397	**9,598**	171.43
	AddrMiner-N	940,256	268,645	3.50
$P_4$	6HAN	**2,845,712**	158,095	18.00
	HMap6-low	2,325,684	9,855	**235.99**
	HMap6-rand	1,480,359	**9,570**	154.69
	AddrMiner-N	945,102	203,686	4.64
$P_5$	6HAN	**3,450,675**	183,547	18.80
	HMap6-low	2,745,667	9,742	**281.80**
	HMap6-rand	1,755,597	**9,148**	191.92
	AddrMiner-N	974,753	263,447	3.70
$P_6$	6HAN	**2,445,186**	132,172	18.50
	HMap6-low	1,855,993	**9,069**	**204.66**
	HMap6-rand	1,490,516	9,935	150.03
	AddrMiner-N	736,914	163,759	4.50

Significant values are in bold.

The accuracy advantage of 6HAN stems from its synergistic mechanism that integrates multi-source feature deep modeling with precise pattern migration. By incorporating heterogeneous graph data, it breaks through feature silos to uncover deep-seated patterns such as consistent maintenance entities and business correlations. Meanwhile, self-supervised dual-task learning enhances the discriminative power of embeddings, ensuring that nonseed prefixes accurately match high-reliability seed patterns. A priority-based target generation strategy allocates resources to patterns with high survival probabilities, thereby avoiding unnecessary resource waste. This tripartite synergy enables 6HAN to focus on high-value addresses within probe budgets. In contrast, the random probing or limited-range exploration of HMap6, as well as the inadequate target-generation associations by AddrMiner-N, result in persistently low hit rates.

In addition, we statistically analyze the time consumption and detection rates of each algorithm in our experiments, as shown in Table 9. Among all seed address sets ($P_1$ to $P_6$), it detected the most addresses, reaching 3,450,675 in $P_5$, significantly outperforming other algorithms. This result validates that 6HAN’s link prediction effectively uncovers deep connections within the IPv6 address structure, thereby generating more highly active detection targets. Regarding temporal efficiency, the almost constant computational overhead due to loading and training the GNN model during execution results in a relatively low detection rate. In contrast, heuristic-based algorithms like HMap6-low, which do not require complex model computations, maintain higher detection rates and shorter execution times.

#### Comparison analysis of IPv6 prefix coverage

**Fig. 7:**
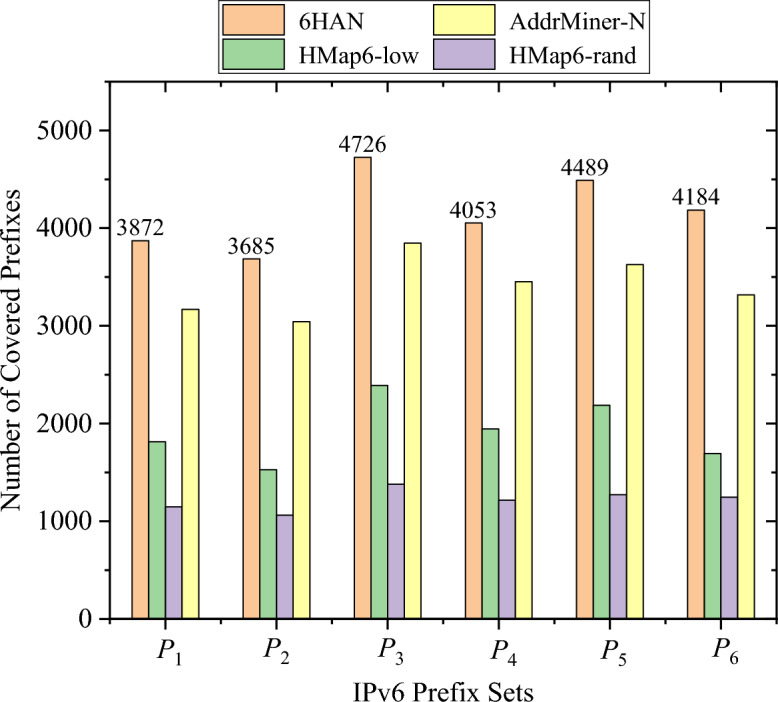
Comparison of IPv6 prefix coverage.

Prefix coverage is evaluated by counting the nonseed prefixes associated with detected IPv6 addresses. As shown in Fig. 7, each algorithm was tested with six seed sets, each with a budget of 50 million probe packets.

Across the six prefix datasets, 6HAN consistently outperforms HMap6 (including HMap6-low and HMap6-rand) and AddrMiner-N in IPv6 prefix coverage. As with hit rate, the large number of probe packets yields narrow confidence intervals. We report them for completeness but emphasize the absolute and relative gains as the primary measures of practical significance. On the $P_3$ prefix set, 6HAN achieves a prefix coverage of 4,726, compared to 2,358 for HMap6-low, 1,323 for HMap6-rand, and 3,742 for AddrMiner-N. This represents absolute gains of 2,368, 3,403, and 984 prefixes, respectively, with corresponding relative improvements of 100.4%, 257.2%, and 26.3%. Across all six datasets, 6HAN improves prefix coverage by 17.41% to 233.58% over baselines. These substantial gains indicate that 6HAN not only detects more active addresses per probe but also discovers them across a broader range of nonseed prefixes, a critical capability for comprehensive IPv6 network mapping.

The prefix coverage advantage of 6HAN originates from its precise pattern matching and equitable significance allocation mechanism. By integrating multi-dimensional features via heterogeneous graph structures, 6HAN adapts seed patterns to a wide range of nonseed prefixes, thereby eliminating coverage blind spots. The 6HAN single-prefix quota mechanism ensures equitable resource allocation to each nonseed prefix, minimizing the neglect of individual prefixes. HMap6-low depends on low-entropy fixed-position generation patterns, making it challenging to adapt to diverse prefix structures and leading to resource concentration. HMap6-rand generates randomly, with limited specificity. AddrMiner performs a complex organization matching, but does not consider other Whois auxiliary information. All three approaches face coverage limitations arising from inherent constraints.

#### Ablation experiment of whois information completion

**Fig. 8:**
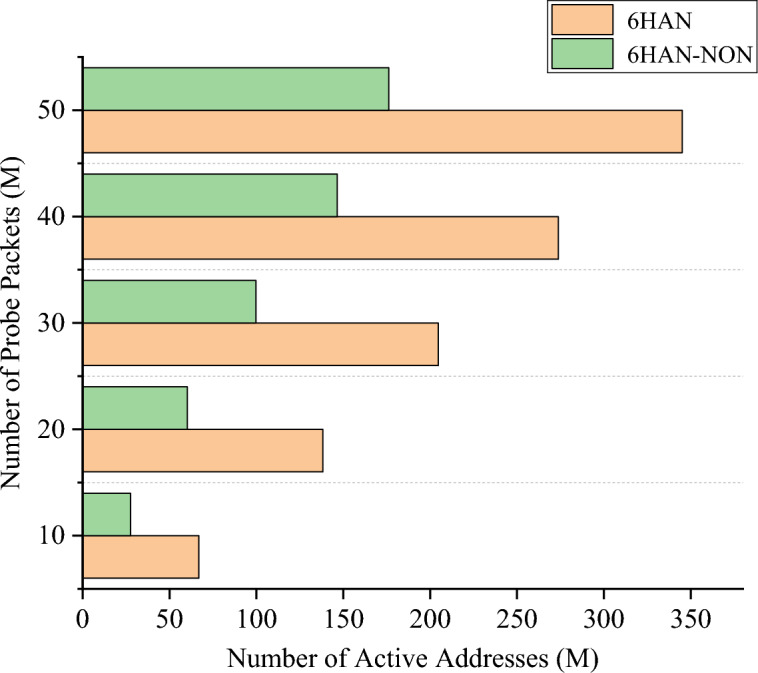
Number of active target addresses detected by 6HAN and 6HAN-NON.

Figure 8 illustrates that with different volumes of probe packets sent, 6HAN (integrated with the Whois information completion module) consistently outperforms 6HAN-NON (the variant without the completion module) in the number of active IPv6 addresses detected, with non-overlapping 95% CIs confirming statistical significance ($p<0.05$). When the number of probe packets reaches 50 million, 6HAN detects 3,450,675 active addresses with a 95% CI of [3447034, 3454316], while 6HAN-NON detects 17,615,78 active IPv6 addresses with a 95% CI of [1758977, 1764179]. The complete non-overlap of the two CIs verifies that the performance gap is not due to random variation. Under identical conditions, 6HAN achieves a 95.88% improvement compared to 6HAN-NON.

The Whois information completion module significantly boosts 6HAN’s performance. Enriching nonseed prefixes with attributes such as maintainer and network name enables precise, multifaceted matching with seed prefixes. This process filters out pseudo-similar seeds (e.g. those with similar embeddings but different maintainers). It aligns migrated patterns with the operational context of target networks, thereby increasing the survival probability of generated addresses.

In contrast, 6HAN-NON, which lacks this module, is limited by incomplete Whois data. Its over-reliance on embeddings alone causes critical attribute mismatches, generating massive invalid addresses and wasting scanning resources. This confirms that the module is essential for improving 6HAN’s resource utilization and detection efficiency.

#### Ablation experiment on semantic-level attention

To evaluate the necessity of removing the semantic-level attention layer, we compared the proposed 6HAN with a variant that retains the full HAN architecture (6HAN-w/sem). As shown in Table 10, both models achieve comparable address hit rates and prefix coverage on the $P_5$ test set, with differences well within the 95% confidence intervals. However, 6HAN reduces time cost by 38.02% compared to 6HAN-w/sem. These results empirically validate our design choice: the semantic-level attention layer is redundant for the IPv6 prefix domain due to the one-to-one nature of prefix-auxiliary relationships, and its removal yields significant efficiency gains without sacrificing detection performance.

**Table 10:** Comparison between 6HAN and the variant with semantic-level attention on the $P_5$ dataset (50M probe packets).

Model	Hit rate (%)	Prefix coverage	Training time (s)
6HAN-proposed	6.46 ± 0.02	4,426 ± 118	188,615
6HAN-w/sem	6.53 ± 0.02	4,421 ± 115	259,327
Improvement	-0.07%	0.1%	38.02%

## Conclusion

To tackle the limitations of existing IPv6 target generation algorithms, including overreliance on unimodal data and inadequate handling of incomplete Whois information, we propose 6HAN, a novel heterogeneous graph attention network-based algorithm. 6HAN constructs a heterogeneous graph by integrating IPv6 prefixes with Whois auxiliary attributes. It employs an attention mechanism to model multi-modal associations and, through dual-task self-supervised training, generates embeddings rich in both semantic discriminability and structural consistency. The algorithm also features a GNN-based Whois completion module and a total budget generation strategy to address the lack of detection efficiency. Overlap between experiments demonstrates that 6HAN significantly outperforms existing methods, achieving 13.81%–201.05% higher hit rates and 17.41%–233.58% greater prefix coverage.

Future research will focus on two directions:

First, we will further expand the dimensions and accuracy of Whois information completion by integrating cross-modal data (e.g. DNS query logs, routing table records, network traffic statistics) with traditional Whois attributes. This integration enriches such a semantic characterization of IPv6 prefixes, while multifaceted, one-shot learning, and meta-learning techniques enhance the model’s adaptability to rare business scenarios and scarce maintenance entity data.

Second, we will optimize the Graph Neural Network architecture and training efficiency to support ultra-large-scale deployment. In the current implementation, which uses approximately 200,000 seed prefixes, the heterogeneous graph comprises roughly 1 million nodes and 2.4 million edges, with training requiring 3.2 GB of memory and 3.2 hours for 100 epochs. While this scale is sufficient to validate the core mechanisms of 6HAN, practical IPv6 deployment may involve hundreds of millions of prefixes, presenting scalability challenges. To address these, we plan to adopt mini-batch training strategies (e.g. GraphSAGE^37^, PinSAGE^38^) to reduce per-epoch computation, and to explore efficient attention mechanisms (e.g. sparse attention, linear attention) to replace fully connected attention, thereby accelerating both training and inference on large-scale graphs.

## Data Availability

The data that support the findings of this study are available from the corresponding author, upon reasonable request.
